# Supplementary material for: Modeling and measuring how codon usage modulates the relationship between burden and yield during protein overexpression in bacteria
Source: bioRxiv. 2024 Nov 28:2024.11.28.625058. Preprint. [Version 1] doi: 10.1101/2024.11.28.625058 (PMC11623672; doi:10.1101/2024.11.28.625058)
Supplement: Supplement 1 [file media-1.pdf]

Supporting Information

Modeling and measuring how codon usage modulates the relationship between burden and yield during protein overexpression in bacteria

Cameron T. Roots[1*], Alexis M. Hill[2*], Claus O. Wilke[2], Jeffrey E. Barrick[1†]

[1] Department of Molecular Biosciences, Center for Systems and Synthetic Biology, The University of Texas at Austin, Austin, Texas, USA [2] Department of Integrative Biology, The University of Texas at Austin, Austin, Texas, USA *Equal contributions †Correspondence: jbarrick@cm.utexas.edu

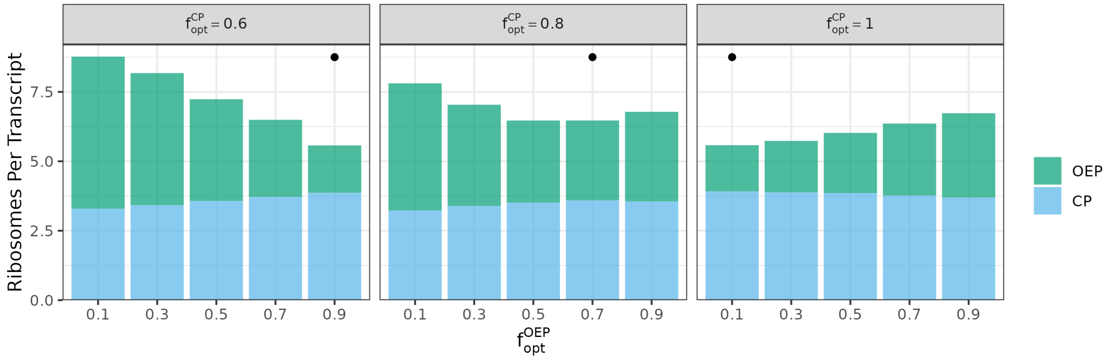

Supplementary Figure S1: **Ribosomes per transcript in overexpression simulations.** Data is for simulations in which the rate of OEP translation initiation is 3 times that of CP initiation. Points indicate the optimal codon fraction in OEP ($f_{\text{opt}}^{\text{OEP}}$) that minimizes cell burden.

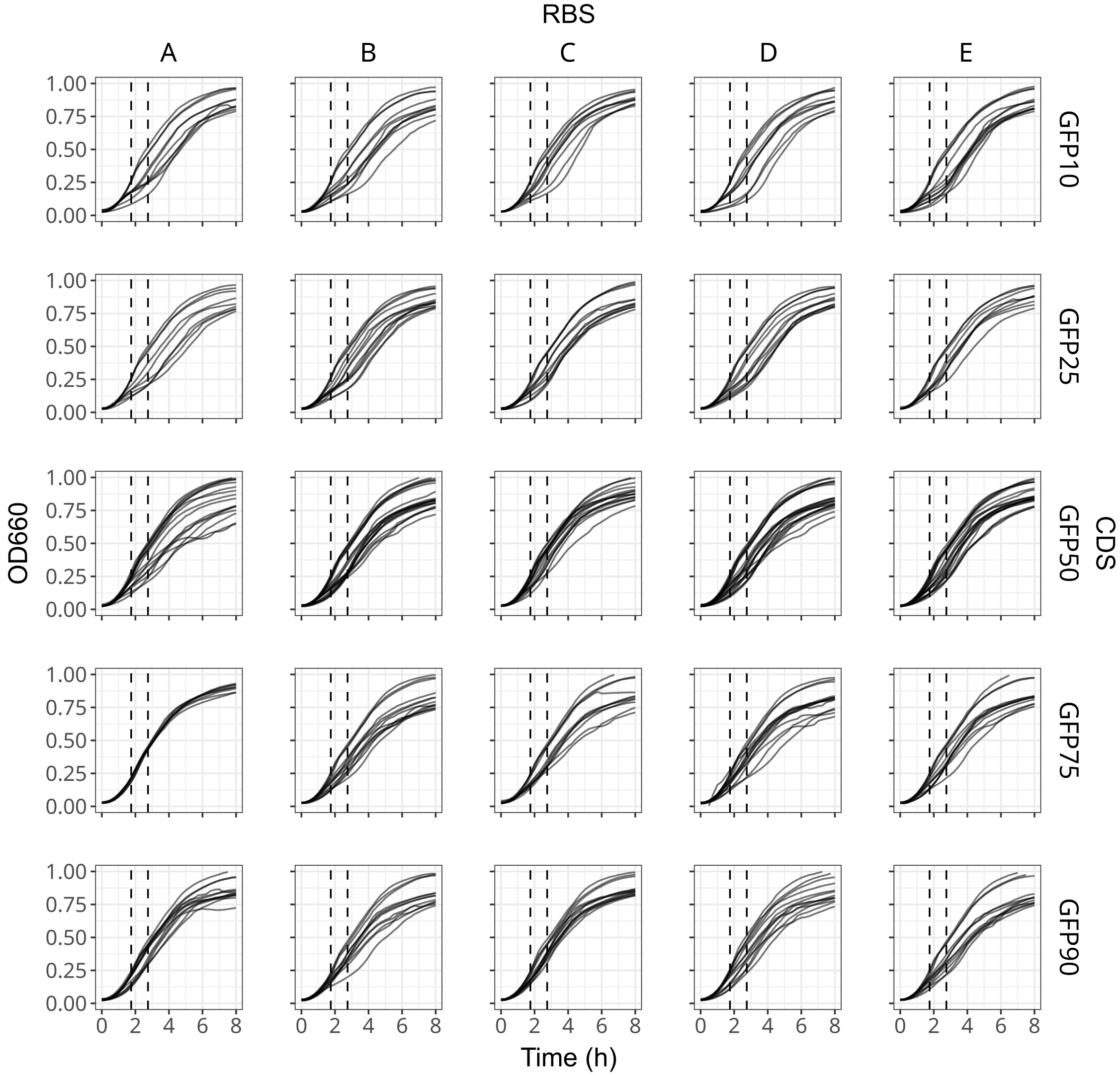

Supplementary Figure S2: **Growth curves of sfGFP constructs engineered to a certain $f_{\mathbf{opt}}$. Vertical dashed lines indicate the window used for fitting the growth rate.**

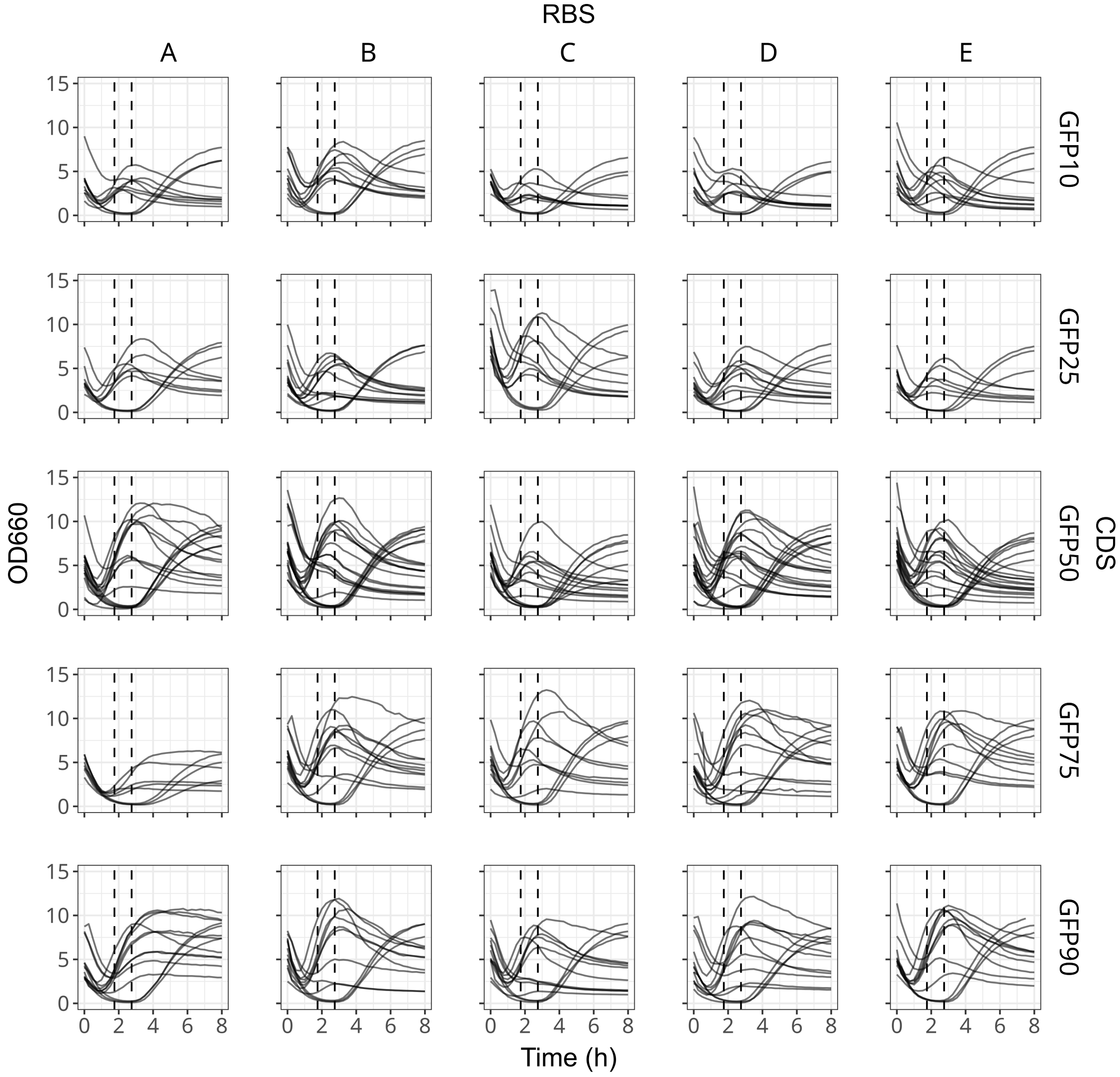

Supplementary Figure S3: **Fluorescence expression curves of sfGFP constructs engineered to a certain $f_{\mathbf{opt}}$.** Vertical dashed lines indicate the window used for fitting the fluorescent protein expression rate.

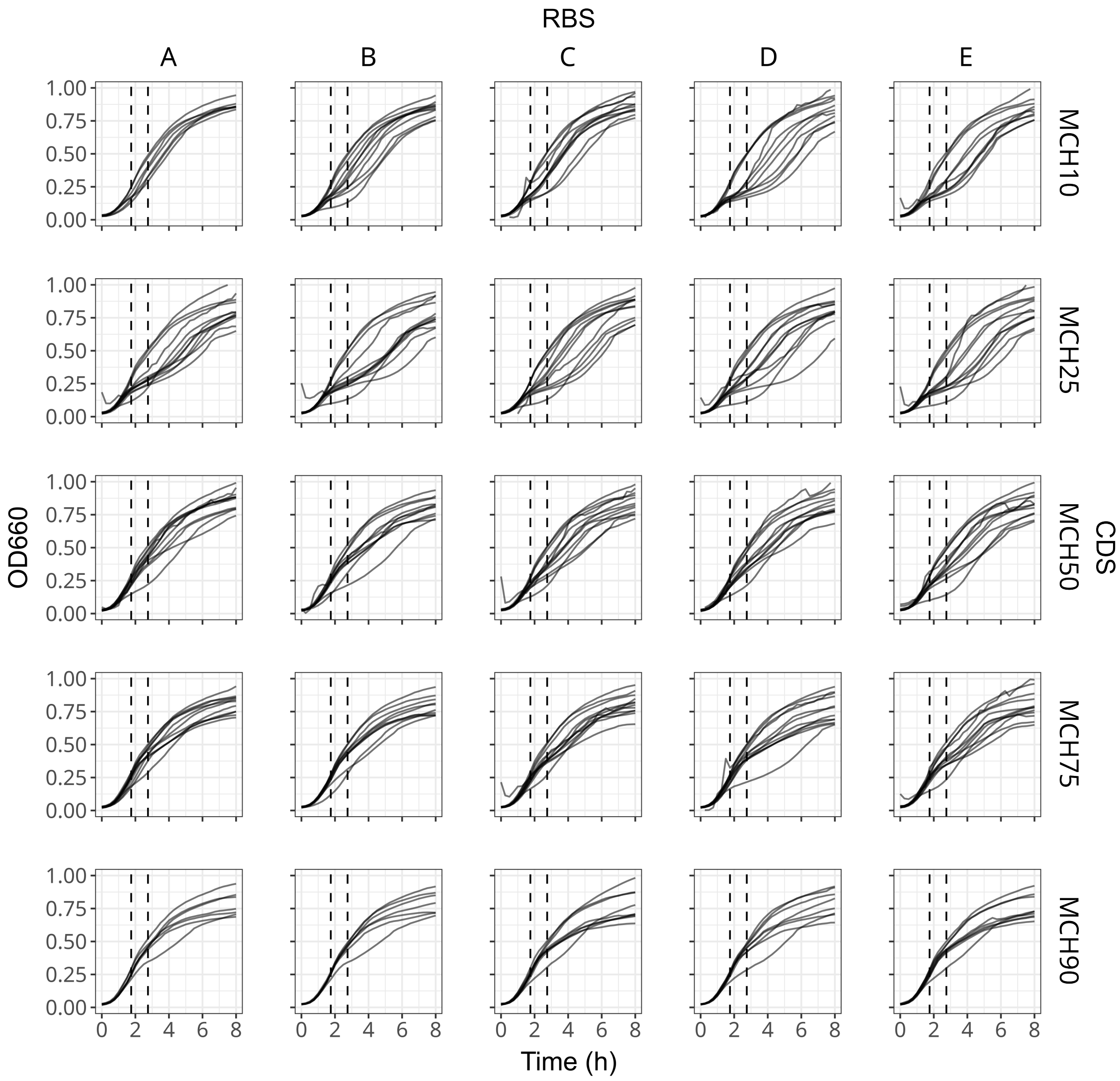

Supplementary Figure S4: **Growth curves of mCherry2 constructs engineered to a certain $f_{\mathbf{opt}}$.** Vertical dashed lines indicate the window used for fitting the growth rate.

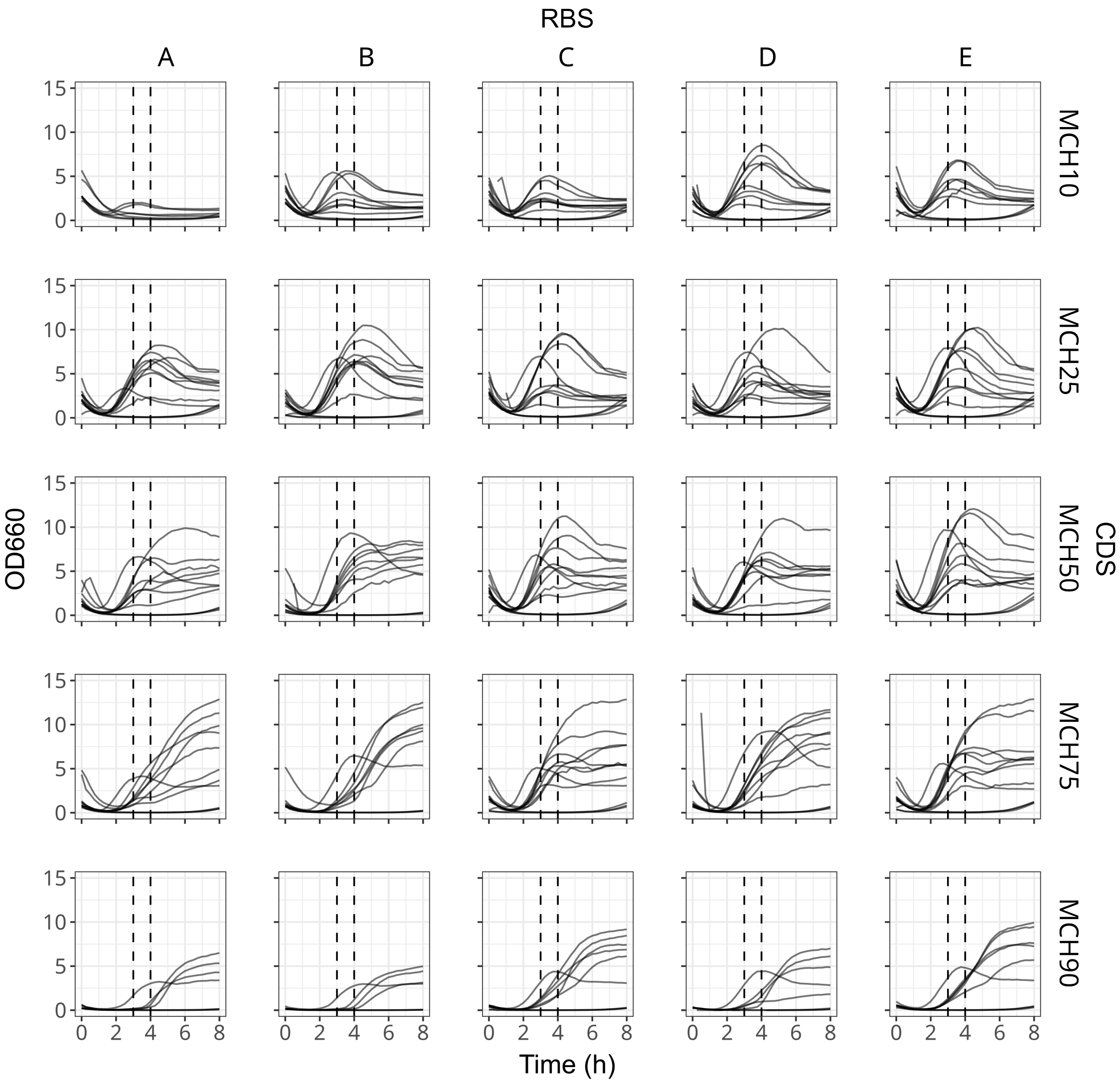

Supplementary Figure S5: **Fluorescence expression curves of mCherry2 constructs engineered to a certain $f_{\mathbf{opt}}$.** Vertical dashed lines indicate the window used for fitting the fluorescent protein expression rate.

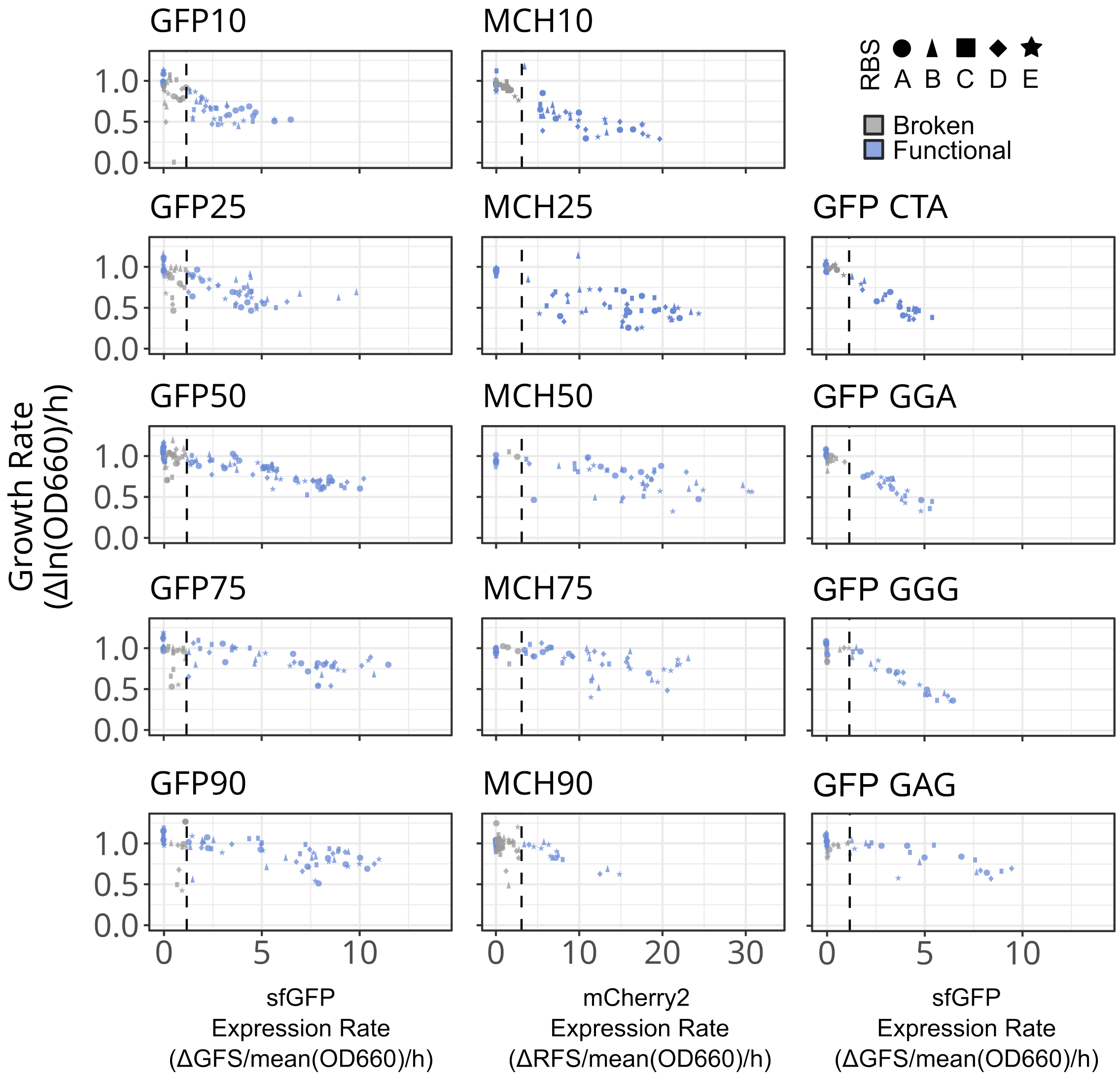

Supplementary Figure S6: **Rates of protein expression versus *E. coli* growth rates for each CDS.** Vertical line represents 10% of the observed maximum expression for each fluorescent protein. Induced constructs below this threshold are presumed to be broken by the time of measurement.

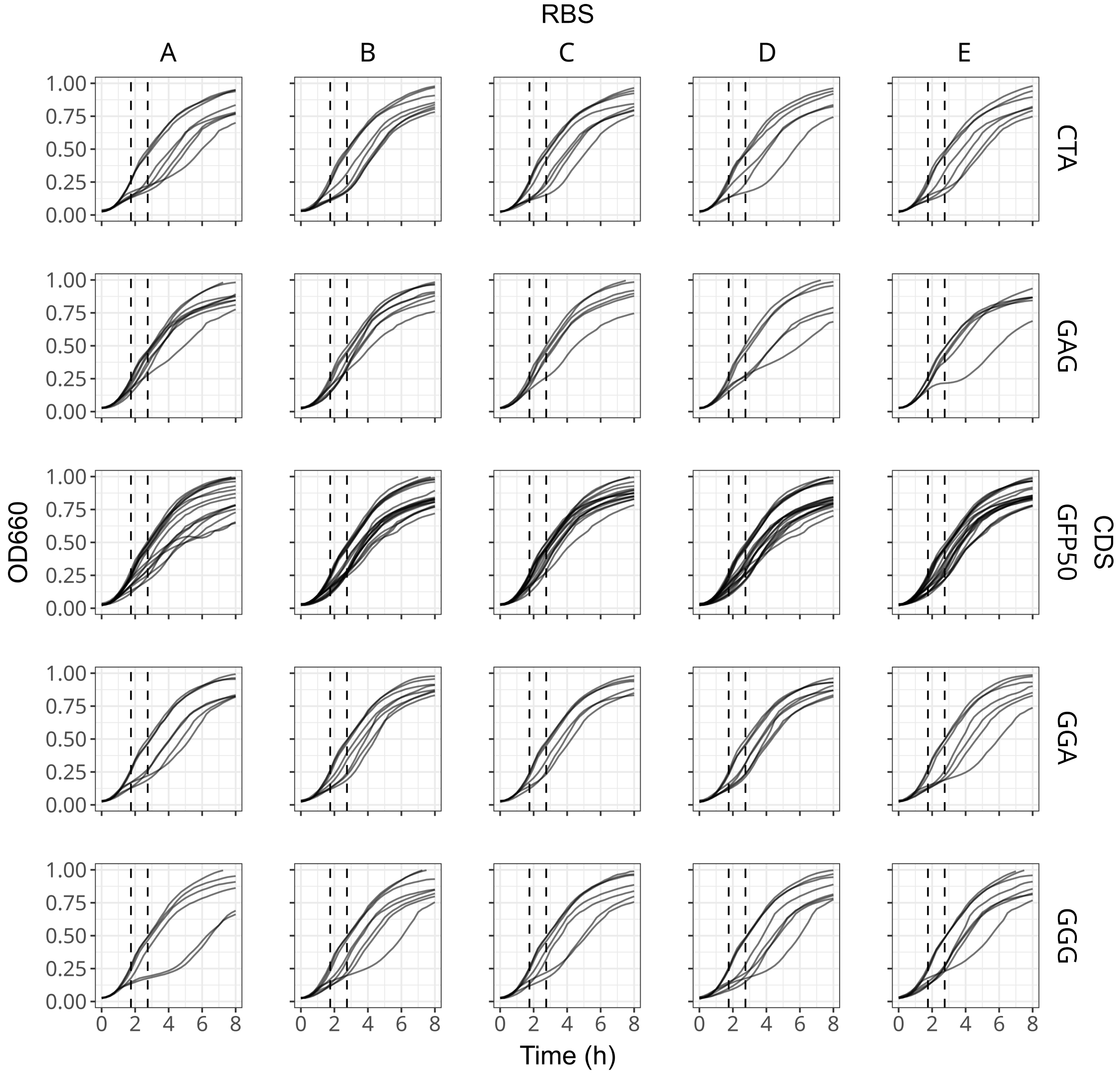

Supplementary Figure S7: **Growth curves of sfGFP constructs where one amino acid was maximally encoded by a rare codon.** Vertical dashed lines indicate the window used for fitting the growth rate.

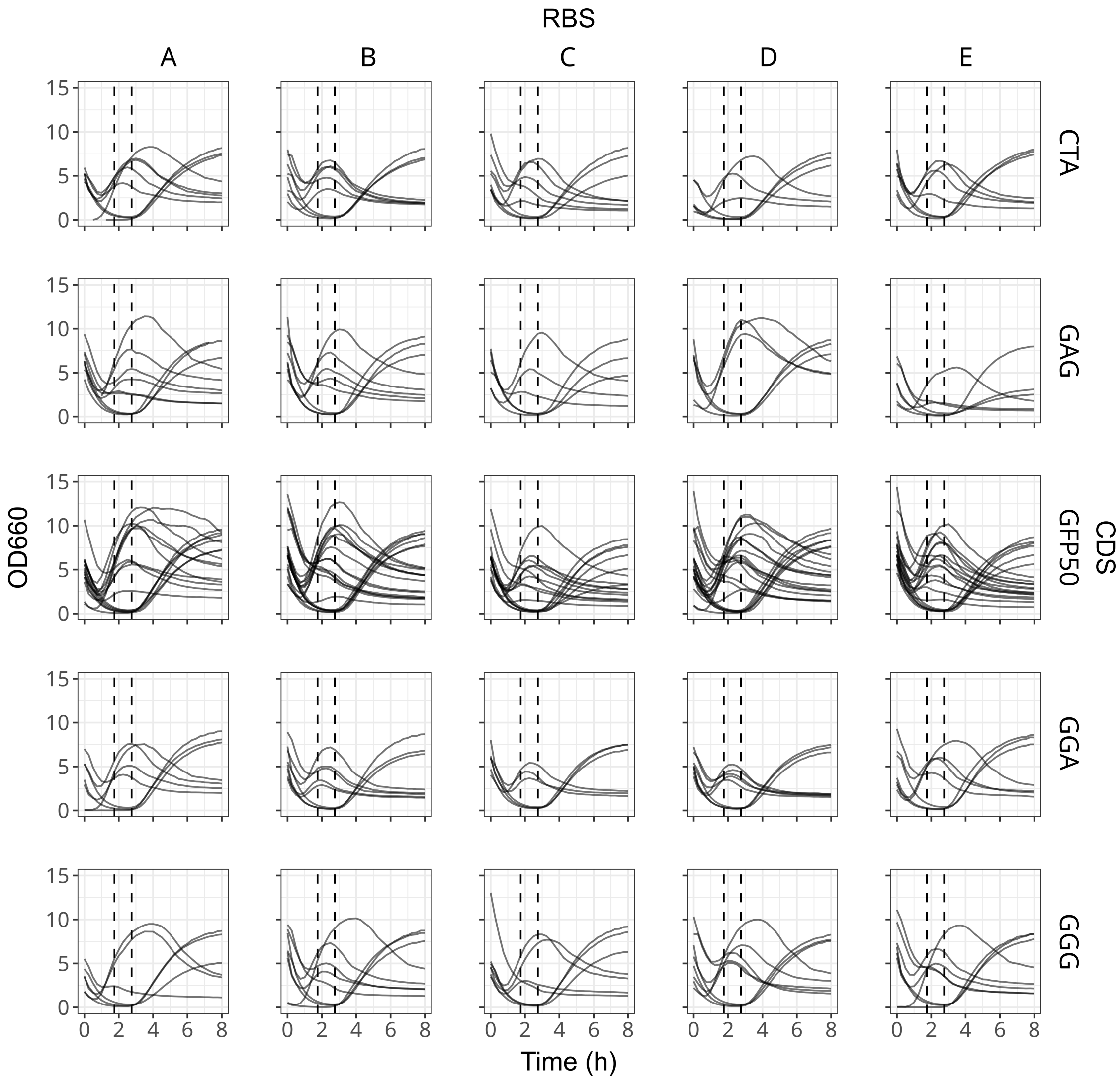

Supplementary Figure S8: **Fluorescence expression curves of codon saturated sfGFP constructs where one amino acid was maximally encoded by a rare codon.** Vertical dashed lines indicate the window used fitting the fluorescent protein expression rate.

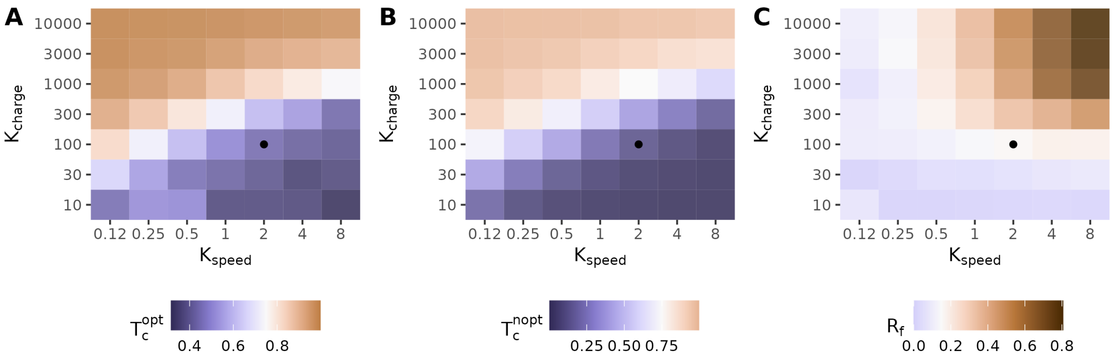

Supplementary Figure S9: **Calibration of tRNA and ribosome steady-states.** Steady-state levels of charged optimal tRNA (A), charged non-optimal tRNA (B), and free (unbound) ribosomes (C) for different charging rate (K_{charge}) and ribosome speed (K_{speed}) parameter values. Steady-states quantities are given as fractions (i.e., as the numbers of charged tRNA molecules or unbound ribosomes divided by total amounts for each species in the simulation). Color scale midpoints are the steady-states observed in real cells growing exponentially, where tRNAs are about 75% charged, and ribosomes are about 20% unbound. Overexpression simulations use a ribosome speed of 2 and a charging rate of 100; the corresponding tiles here have been annotated with black points.

Supplementary Table S1: **Growth rate and expression parameters fit for all constructs**

CDS	Maximum Expression Rate (ΔFS/mean(OD660)/h)	Growth Rate at Maximum Expression (Δln(OD660)/h)	Fit Intercept	Fit Slope ($-$Overexpression Burden Cost)	Calculated Burden at Maximum Expression
GFP10	5.42	0.55	1.02	-0.115	61%
GFP25	7.54	0.619	1.02	-0.072	54%
GFP50	9.10	0.68	1.02	-0.040	36%
GFP75	10.66	0.81	1.02	-0.027	29%
GFP90	10.26	0.79	1.02	-0.024	24%
MCH10	18.27	0.37	0.95	-0.037	71%
MCH25	22.38	0.46	0.95	-0.027	64%
MCH50	27.98	0.58	0.95	-0.013	30%
MCH75	21.94	0.83	0.95	-0.009	22%
MCH90	13.58	0.66	0.95	-0.014	21%
CTA	4.90	0.45	1.04	-0.133	63%
GAG	8.92	0.65	1.04	-0.041	35%
GGA	5.17	0.43	1.04	-0.122	61%
GGG	6.33	0.39	1.04	-0.102	63%

Supplementary Table S2: **Primers used in this study**

Name	Purpose	Sequence
T7tag_F	Remove T7 tag from pET21b(+)	CATATGTATATCTCCTTCTTAAAGTTAAACAAAATTATTTCTAGAGGGGAATTGTTATCC
T7tag_R	Remove T7 tag from pET21b(+)	GATCCGAATTCGAGCTCCGT
Histag_F	Remove His tag from pET21b(+)	TTATTATTACTCGAGTGCGGCCGCAA
Histag_R	Remove His tag from pET21b(+)	TGAGATCCGGCTGCTAACAAAGCC
pET21_F1	Make PaqCI domesticated pET21 Dropout Vector	TTACGCGGTCTCGAGCCACGCTCACCGGCTCCAG
pET21_R1	Make PaqCI domesticated pET21 Dropout Vector	TTACGCGGTCTCGACAAAGCCCGAAAGGAAGCTGAGTTGG
pET21_F2	Make PaqCI domesticated pET21 Dropout Vector	TTACGCGGTCTCGACGCCGGACGCATCGTGGCCG
pET21_R2	Make PaqCI domesticated pET21 Dropout Vector	TTACGCGGTCTCGGGCTCTCGCGGAATCATTGCAGCACTG
pBTK1001_F	Make PaqCI domesticated pET21 Dropout Vector	TTACGCGGTCTCGTTGTTATTACGCAGGTGGAAAGTGAAACGTG
pBTK1001_R	Make PaqCI domesticated pET21 Dropout Vector	TTACGCGGTCTCGGCGTATCCAGCAGGTGTATAAACGCAG
GFP_F	Assembling GFP Parts	TTACGCGGTCTCGTTGTTATTACGCAGGTGGAAAGTGAAACGTGATTTCATG
GFP_R	Assembling GFP Parts	TTACGCGGTCTCGGCGTATCCAGCAGGTGTATAAACGCAGAAAGGCC
MCH_F	Assemblying mCherry Parts	ATGCCGTCTCATCGGCACCTGCTATACATATGGTGAGCAAGGGCGAG
MCH_R	Assembling mCherry Parts	GCATCGTCTCAGGTCACCTGCCTTTGTTAGCAGCCGGATCTCATTATTA
RBS.A_F	Assembling RBS.A Part	GCATCGTCTCAGGTCACCTGCACCATATGTATAACTCCTTCTTAAAGTTAAACAAAAT
RBS.B_F	Assembling RBS.B Part	GCATCGTCTCAGGTCACCTGCACCATATGTATATATCCTTCTTAAAGTTAAACAAAAT
RBS.C_F	Assembling RBS.C Part	GCATCGTCTCAGGTCACCTGCACCATATGTATATCTCCTTCTTAAAGTTAAATAAAAT
RBS.D_F	Assembling RBS.D Part	GCATCGTCTCAGGTCACCTGCACCATATGTATATCTCCTCCTTAAAGTTAAACAAAAT
RBS.E_F	Assembling RBS.E Part	GCATCGTCTCAGGTCACCTGCACCATATGTATATCTCCTTCTTAAAGTTAAACAAAAT
RBS_R	Assembling RBS Parts	ATGCCGTCTCATCGGCACCTGCCCGGCGTAGAGGATCGAGATC

Supplementary Table S3: **Plasmids Used in this study**

Plasmid	Source	Plasmid	Source
pET21b(+)	Novagen	pET21-Domesticated	This study
pBTK1001	Lariviere 2024		
pBTK1001-sfGFP.10	This study	pET21-RBS.A-sfGFP.GGG	This study
pBTK1001-sfGFP.25	This study	pET21-RBS.B-sfGFP.GGG	This study
pBTK1001-sfGFP.50	This study	pET21-RBS.C-sfGFP.GGG	This study
pBTK1001-sfGFP.75	This study	pET21-RBS.D-sfGFP.GGG	This study
pBTK1001-sfGFP.90	This study	pET21-RBS.E-sfGFP.GGG	This study
pBTK1001-sfGFP.GGG	This study	pET21-RBS.A-sfGFP.GGA	This study
pBTK1001-sfGFP.GGA	This study	pET21-RBS.B-sfGFP.GGA	This study
pBTK1001-sfGFP.GAG	This study	pET21-RBS.C-sfGFP.GGA	This study
pBTK1001-sfGFP.CTA	This study	pET21-RBS.D-sfGFP.GGA	This study
		pET21-RBS.E-sfGFP.GGA	This study
pBTK1001-mCherry2.10	This study	pET21-RBS.A-sfGFP.GAG	This study
pBTK1001-mCherry2.25	This study	pET21-RBS.B-sfGFP.GAG	This study
pBTK1001-mCherry2.50	This study	pET21-RBS.C-sfGFP.GAG	This study
pBTK1001-mCherry2.75	This study	pET21-RBS.D-sfGFP.GAG	This study
pBTK1001-mCherry2.90	This study	pET21-RBS.E-sfGFP.GAG	This study
pBTK1001-RBS.A	This study	pET21-RBS.A-sfGFP.CTA	This study
pBTK1001-RBS.B	This study	pET21-RBS.B-sfGFP.CTA	This study
pBTK1001-RBS.C	This study	pET21-RBS.C-sfGFP.CTA	This study
pBTK1001-RBS.D	This study	pET21-RBS.D-sfGFP.CTA	This study
pBTK1001-RBS.E	This study	pET21-RBS.E-sfGFP.CTA	This study
pET21-RBS.A-sfGFP.10	This study	pET21-RBS.A-mCherry2.10	This study
pET21-RBS.B-sfGFP.10	This study	pET21-RBS.B-mCherry2.10	This study
pET21-RBS.C-sfGFP.10	This study	pET21-RBS.C-mCherry2.10	This study
pET21-RBS.D-sfGFP.10	This study	pET21-RBS.D-mCherry2.10	This study
pET21-RBS.E-sfGFP.10	This study	pET21-RBS.E-mCherry2.10	This study
pET21-RBS.A-sfGFP.25	This study	pET21-RBS.A-mCherry2.25	This study
pET21-RBS.B-sfGFP.25	This study	pET21-RBS.B-mCherry2.25	This study
pET21-RBS.C-sfGFP.25	This study	pET21-RBS.C-mCherry2.25	This study
pET21-RBS.D-sfGFP.25	This study	pET21-RBS.D-mCherry2.25	This study
pET21-RBS.E-sfGFP.25	This study	pET21-RBS.E-mCherry2.25	This study
pET21-RBS.A-sfGFP.50	This study	pET21-RBS.A-mCherry2.50	This study
pET21-RBS.B-sfGFP.50	This study	pET21-RBS.B-mCherry2.50	This study
pET21-RBS.C-sfGFP.50	This study	pET21-RBS.C-mCherry2.50	This study
pET21-RBS.D-sfGFP.50	This study	pET21-RBS.D-mCherry2.50	This study
pET21-RBS.E-sfGFP.50	This study	pET21-RBS.E-mCherry2.50	This study
pET21-RBS.A-sfGFP.75	This study	pET21-RBS.A-mCherry2.75	This study
pET21-RBS.B-sfGFP.75	This study	pET21-RBS.B-mCherry2.75	This study
pET21-RBS.C-sfGFP.75	This study	pET21-RBS.C-mCherry2.75	This study
pET21-RBS.D-sfGFP.75	This study	pET21-RBS.D-mCherry2.75	This study
pET21-RBS.E-sfGFP.75	This study	pET21-RBS.E-mCherry2.75	This study
pET21-RBS.A-sfGFP.90	This study	pET21-RBS.A-mCherry2.90	This study
pET21-RBS.B-sfGFP.90	This study	pET21-RBS.B-mCherry2.90	This study
pET21-RBS.C-sfGFP.90	This study	pET21-RBS.C-mCherry2.90	This study
pET21-RBS.D-sfGFP.90	This study	pET21-RBS.D-mCherry2.90	This study
pET21-RBS.E-sfGFP.90	This study	pET21-RBS.E-mCherry2.90	This study

Supplementary Table S4: **Sequences of sfGFP genes with different codon usage**

**CDS Name**	**Nucleotide Sequence**
GFP10	ATGAGCAAAGGTGAAGAACTGTTTACCGGCGTTGTGCCGATTCTGGTGGAGCTAGATGGGGATGTGAAT<br>GGACATAAATTTAGCGTGCGGGGGGAGGGAGAAGGGGATGCGACGAATGGGAAACTTACATTAAAATT<br>TATTTGTACAACGGGAAAACTCCCTGTCCCATGGCCTACATTGGTGACGACGCTAACATATGGAGTCCA<br>ATGTTTTAGCCGGTATCCCGATCATATGAAACGACATGATTTCTTTAAAAGCGCGATGCCCGAGGGATA<br>TGTGCAAGAGAGGACCATTAGCTTTAAAGATGATGGAACATATAAAACACGGGCGGAAGTTAAATTTGA<br>GGGGGATACGCTAGTGAATAGAATTGAGCTAAAAGGTATTGATTTTAAAGAGGATGGGAATATTCTTGG<br>ACATAAACTAGAGTATAATTTCAACAGCCATAATGTGTATATTACAGCCGATAAACAAAAAAATGGGAT<br>AAAAGCGAACTTTAAAATTAGACATAATGTGGAGGATGGGAGCGTGCAACTAGCGGATCATTATCAACA<br>GAATACGCCTATTGGTGATGGCCCCGTGCTGCTTCCCGATAATCATTATCTAAGCACACAAAGCGTGTT<br>GAGCAAAGATCCAAATGAGAAACGGGATCATATGGTGTTGCTCGAATTTGTCACAGCCGCGGGAATTAC<br>ACATGGAATGGATGAGTTGTATAAA
GFP25	ATGAGCAAAGGTGAAGAACTGTTTACCGGCGTTGTGCCGATTCTGGTGGAACTTGATGGTGATGTGAAT<br>GGGCATAAATTTAGCGTCAGGGGCGAAGGAGAGGGTGATGCGACGAACGGAAAACTGACGCTGAAATT<br>TATTTGTACCACCGGTAAACTTCCGGTCCCATGGCCCACCCTCGTGACCACACTAACCTATGGAGTCCAA<br>TGTTTTAGCCGCTATCCTGATCATATGAAACGCCATGATTTTTTTAAAAGCGCGATGCCTGAGGGCTAT<br>GTGCAAGAGCGTACCATTAGCTTTAAAGATGATGGAACCTATAAAACCCGAGCGGAAGTCAAATTTGAG<br>GGCGATACCCTAGTGAACAGAATTGAGCTGAAAGGAATTGATTTTAAAGAGGATGGCAATATTTTAGGG<br>CATAAACTCGAGTATAATTTCAACAGCCATAATGTGTATATTACCGCCGATAAACAAAAAAATGGAATC<br>AAAGCGAACTTTAAAATTAGGCACAACGTGGAAGATGGAAGCGTGCAACTTGCGGATCATTATCAGCAG<br>AATACACCGATTGGTGATGGCCCAGTGCTCCTCCCAGATAATCATTATCTTAGCACCCAGAGCGTCCTGA<br>GCAAAGATCCAAATGAGAAAAGGGATCATATGGTGCTGCTTGAGTTTGTTACCGCCGCGGGGATTACGC<br>ACGGAATGGATGAGTTATATAAA
GFP50	ATGAGCAAAGGTGAAGAACTGTTTACCGGCGTTGTGCCGATTCTGGTGGAACTGGATGGTGATGTGAAT<br>GGCCATAAATTTAGCGTTCGTGGCGAAGGAGAAGGAGATGCGACCAACGGTAAACTGACCCTGAAATTT<br>ATTTGCACCACCGGTAAACTGCCGGTTCCGTGGCCCACCCTGGTGACCACCCTGACCTATGGCGTTCAGT<br>GTTTTAGCCGCTATCCGGATCATATGAAACGCCATGATTTCTTTAAAAGCGCGATGCCGGAAGGCTATG<br>TGCAGGAACGTACCATTAGCTTCAAAGATGATGGCACCTATAAAACCCGTGCGGAGGTGAAATTTGAAG<br>GCGATACCCTCGTGAACCGCATTGAACTGAAAGGTATTGATTTTAAAGAAGATGGCAATATTCTGGGTC<br>ATAAACTGGAATATAATTTTAACAGCCATAATGTGTATATTACCGCCGATAAACAGAAAAATGGCATCA<br>AAGCGAATTTTAAAATCCGTCACAACGTGGAGGATGGTAGCGTGCAGTTAGCGGATCATTATCAGCAGA<br>ATACCCCGATTGGTGATGGCCCGGTGCTGCTGCCGGATAATCATTATCTGAGCACCCAAAGCGTTCTGA<br>GCAAAGATCCGAATGAAAAACGTGATCATATGGTGCTGCTGGAATTTGTTACCGCCGCGGGCATTACCC<br>ACGGTATGGATGAACTGTATAAA
GFP75	ATGAGCAAAGGTGAAGAACTGTTTACCGGCGTTGTGCCGATTCTGGTGGAACTGGATGGTGATGTTAAC<br>GGCCACAAATTCAGCGTTCGTGGCGAAGGCGAAGGTGACGCTACCAACGGTAAACTGACCCTGAAATTT<br>ATCTGCACCACCGGTAAACTGCCGGTTCCGTGGCCGACCCTGGTGACCACCCTGACCTATGGCGTTCAG<br>TGCTTCAGCCGCTACCCGGACCATATGAAACGCCATGACTTCTTCAAAAGCGCGATGCCGGAAGGCTAT<br>GTGCAGGAACGTACCATTAGCTTCAAAGATGACGGCACCTATAAAACCCGTGCGGAAGTTAAATTCGAA<br>GGCGATACCCTGGTTAACCGCATCGAACTGAAAGGTATCGATTTTAAAGAAGACGGCAACATCCTGGGT<br>CATAAACTGGAATATAACTTCAACAGCCACAATGTTTATATTACCGCCGATAAACAGAAAAACGGCATCA<br>AAGCGAACTTTAAAATCCGTCACAACGTAGAAGATGGTTCTGTGCAGCTGGCGGATCATTATCAGCAGA<br>ACACCCCGATCGGTGATGGCCCGGTACTGCTGCCGGATAATCATTACCTGAGCACCCAGTCCGTTCTGT<br>CTAAAGATCCGAATGAAAAACGTGACCATATGGTGCTGCTGGAATTTGTTACCGCTGCTGGCATCACCC<br>ACGGTATGGACGAACTGTACAAA
GFP90	ATGAGCAAAGGTGAAGAACTGTTTACCGGCGTTGTGCCGATTCTGGTGGAACTGGACGGTGATGTTAAC<br>GGCCACAAATTCAGCGTTCGTGGCGAAGGCGAAGGTGACGCTACCAACGGTAAACTGACCCTGAAATTC<br>ATCTGCACCACCGGTAAACTGCCGGTTCCGTGGCCGACCCTGGTAACCACCCTGACCTATGGCGTTCAG<br>TGCTTCAGCCGCTACCCGGACCACATGAAACGCCACGACTTCTTCAAATCTGCTATGCCGGAAGGCTATG<br>TACAGGAACGTACCATCAGCTTCAAAGACGACGGCACCTACAAAACCCGTGCGGAAGTTAAATTCGAAG<br>GCGACACCCTGGTTAACCGCATCGAACTGAAAGGTATCGATTTCAAAGAAGACGGCAACATCCTGGGTC<br>ACAAACTGGAATACAACTTCAACTCTCACAACGTTTACATTACCGCCGACAAACAGAAAAACGGCATCAA<br>AGCTAACTTCAAAATCCGTCACAACGTAGAAGACGGTTCTGTACAGCTGGCGGACCATTACCAGCAGAA<br>CACCCCGATCGGTGATGGCCCGGTACTGCTGCCGGACAACCACTACCTGAGCACCCAGTCCGTTCTGTCT<br>AAAGATCCGAACGAAAAACGTGACCACATGGTTCTGCTGGAATTTGTTACCGCTGCTGGCATCACCCAC<br>GGTATGGACGAACTGTACAAA

Supplementary Table S5: **Sequences of mCherry2 genes with different codon usage**

CDS Name	Nucleotide Sequence
MCH10	ATGGTGAGCAAGGGCGAGGAGGATAACCTGGCCATCATCAAGGAGTTTATGCGGTTTAAGGTGCATATG<br>GAGGGGAGTGTGAATGGACATGAGTTCGAGATCGAGGGGGAGGGAGAGGGCAGACCCTATGAGGGAAC<br>ACAAACGGCCAAGTTGAAGGTGACGAAGGGTGGACCCTTGCCCTTTGCCTGGGATATTCTCAGTCCTCA<br>ATTTATGTATGGGAGCAAGGCCTATGTGAAGCATCCCGCCGACATACCCGATTACTTGAAGTTGAGTTT<br>TCCCGAGGGGTTTAATTGGGAGAGGGTGATGAATTTTGAGGATGGCGGGGTGGTGACCGTGACCCAGG<br>ATTCAAGTCTTCAAGATGGCGAGTTTATATATAAGGTGAAGTTACGAGGGACAAATTTTCCCAGCGATG<br>GACCCGTAATGCAATGTCGAACCATGGGGTGGGAGGCCTCGACAGAGCGGATGTACCCCGAGGATGGAG<br>CCTTGAAGGGAGAGATTAAGCAAAGGCTTAAGTTAAAGGATGGGGGACATTATGATGCCGAGGTCAAGA<br>CGACATATAAGGCCAAGAAGCCCGTGCAACTACCCGGAGCCTATAATGTCGATATAAAGTTGGATATAC<br>TTTCGCATAATGAGGACTATACGATTGTGGAACAATATGAGCGGGCCGAGGGGCGGCATAGCACGGGAG<br>GGATGGATGAGCTTTACAAG
MCH25	ATGGTGAGCAAGGGCGAGGAGGATAACCTGGCCATCATCAAGGAGTTCATGAGATTTAAGGTGCATATG<br>GAGGGCTCAGTGAATGGCCACGAGTTTGAGATTGAGGGCGAGGGCGAGGGGCGCCCCTATGAGGGCAC<br>ACAAACAGCCAAGTTGAAGGTGACCAAGGGAGGGCCCTTACCCTTCGCCTGGGATATATTGTCACCTCA<br>ATTTATGTATGGGTCGAAGGCCTATGTGAAGCACCCCGCCGATATCCCCGACTACTTGAAGCTGTCATT<br>TCCCGAGGGGTTTAATTGGGAGCGGGTGATGAATTTCGAGGACGGCGGGGTGGTGACCGTGACCCAAG<br>ATAGTAGTCTGCAAGATGGCGAGTTTATATATAAGGTGAAGTTGAGAGGAACCAATTTCCCCTCGGACG<br>GCCCCGTGATGCAATGTAGGACAATGGGCTGGGAGGCCAGCACTGAGCGGATGTATCCCGAGGACGGAG<br>CCTTAAAGGGAGAGATCAAGCAGAGGCTGAAGCTTAAGGACGGAGGGCATTACGACGCCGAGGTCAAG<br>ACAACCTACAAGGCCAAGAAGCCCGTGCAATTGCCCGGCGCCTATAATGTCGATATCAAGTTGGATATT<br>CTTTCCCACAATGAGGACTATACCATCGTGGAACAGTACGAGCGGGCCGAGGGAAGGCATAGTACAGGC<br>GGCATGGATGAGCTCTATAAG
MCH50	ATGGTGAGCAAGGGCGAGGAGGATAACCTGGCCATCATCAAGGAGTTCATGAGATTCAAGGTGCACATG<br>GAGGGCTCCGTGAACGGCCATGAGTTCGAGATCGAGGGCGAGGGCGAGGGCCGCCCCTATGAGGGCAC<br>ACAGACCGCCAAGCTTAAGGTGACAAAGGGTGGCCCCCTGCCCTTCGCCTGGGACATACTGAGTCCTCA<br>GTTCATGTACGGCTCCAAGGCCTACGTGAAGCACCCCGCCGACATCCCCGACTACTTGAAGCTGTCCTTC<br>CCCGAGGGCTTTAATTGGGAGCGCGTGATGAACTTCGAGGACGGCGGCGTGGTGACCGTGACCCAGGAC<br>TCCTCCCTGCAGGACGGCGAGTTTATCTACAAGGTGAAGCTGCGCGGCACCAACTTCCCCTCCGATGGA<br>CCCGTAATGCAGTGTAGAACAATGGGATGGGAGGCCTCCACAGAGCGGATGTACCCCGAGGACGGCGCC<br>CTGAAGGGGGAGATCAAGCAGAGGTTGAAGCTTAAGGACGGGGGCCACTACGACGCTGAGGTCAAGAC<br>CACCTACAAGGCCAAGAAGCCCGTGCAACTGCCCGGGGCCTACAACGTCGACATCAAGTTGGACATCCT<br>TAGTCACAACGAGGACTACACCATCGTGGAGCAATATGAGCGCGCCGAGGGAAGGCATTCCACCGGCGG<br>CATGGACGAGTTATACAAGTAATAA
MCH75	ATGGTGAGCAAGGGCGAGGAGGATAACCTGGCCATCATCAAGGAGTTCATGCGCTTCAAGGTGCACATG<br>GAGGGCTCCGTGAACGGCCACGAGTTCGAAATCGAAGGCGAGGGCGAGGGCCGCCCCTACGAGGGCACC<br>CAGACCGCCAAGCTGAAGGTGACCAAGGGTGGCCCCCTGCCCTTCGCTTGGGACATCCTGTCCCCTCAG<br>TTCATGTACGGCTCCAAGGCCTACGTGAAGCACCCCGCCGACATCCCCGACTACTTGAAGCTGTCCTTCC<br>CCGAGGGCTTCAATTGGGAGCGCGTAATGAACTTCGAAGACGGCGGCGTGGTGACCGTTACCCAGGACT<br>CCTCCCTGCAGGACGGCGAGTTCATCTACAAGGTGAAGCTGCGCGGCACCAACTTCCCCTCCGACGGCC<br>CCGTAATGCAGTGCCGTACCATGGGCTGGGAAGCTTCCACTGAACGGATGTACCCCGAGGACGGCGCCC<br>TGAAGGGCGAGATCAAGCAGAGGCTGAAGCTGAAGGACGGCGGCCACTACGACGCTGAAGTCAAGACCA<br>CCTACAAGGCCAAGAAGCCCGTTCAGCTGCCGGGCGCCTACAACGTCGACATCAAGCTGGACATCCTTT<br>CCCACAACGAGGACTACACCATCGTGGAACAGTACGAACGCGCTGAAGGCCGCCACTCCACCGGCGGCA<br>TGGACGAACTGTACAAG
MCH90	ATGGTGAGCAAGGGCGAGGAGGATAACCTGGCCATCATCAAGGAATTCATGCGTTTCAAAGTCCACATG<br>GAGGGTTCTGTAAACGGCCACGAATTCGAAATCGAAGGTGAAGGTGAAGGTCGTCCGTACGAAGGCACT<br>CAGACTGCTAAGCTGAAAGTTACCAAAGGCGGTCCGCTGCCGTTCGCTTGGGACATCCTGTCCCCGCAG<br>TTCATGTACGGTTCCAAAGCTTACGTAAAGCACCCGGCCGACATACCGGACTACCTAAAGTTGTCTTTCC<br>CGGAAGGTTTCAACTGGGAACGCGTTATGAACTTCGAAGACGGCGGCGTTGTCACTGTAACTCAGGACT<br>CTTCTTTACAGGACGGTGAATTCATCTACAAGGTAAAGCTGCGCGGTACCAACTTCCCTTCTGACGGTCC<br>GGTAATGCAGTGCCGTACTATGGGCTGGGAAGCTTCTACCGAAAGGATGTACCCGGAAGACGGTGCTCT<br>GAAAGGTGAAATCAAGCAGCGTTTAAAGCTGAAGGACGGCGGCCACTACGACGCTGAAGTTAAGACCAC<br>TTACAAAGCTAAGAAACCGGTTCAGCTGCCGGGCGCTTACAACGTTGACATCAAGCTGGACATCCTGTC<br>TCACAACGAAGACTACACTATCGTTGAACAGTACGAACGTGCTGAAGGTCGTCACAGTACTGGCGGCAT<br>GGACGAATTGTATAAG

Supplementary Table S6: **Sequences of sfGFP genes with one target codon deoptimized**

**CDS Name**	**Nucleotide Sequence**
GFP GGG	ATGAGCAAAGGTGAAGAACTGTTTACCGGCGTTGTGCCGATTCTGGTGGAACTGGATGGGGATGTGAAT
	GGGCACAAATTTAGCGTTCGTGGGGAAGGGGAAGGGGATGCGACCAACGGGAAACTGACCCTGAAATTT
	ATTTGCACCACCGGGAAACTGCCGGTTCCGTGGCCCACCCTGGTGACCACCCTGACCTATGGGGTTCAG
	TGTTTTAGCCGCTATCCGGATCATATGAAACGCCATGATTTCTTTAAAAGCGCGATGCCGGAAGGGTAT
	GTGCAGGAACGTACCATTAGCTTCAAAGATGATGGGACCTATAAAACCCGTGCGGAGGTGAAATTTGAA
	GGGGATACCCTCGTGAACCGCATTGAACTGAAAGGGATTGATTTTAAAGAAGATGGGAATATTCTGGGG
	CATAAACTGGAATATAATTTTAACAGCCATAATGTGTATATTACCGCCGATAAACAGAAAAATGGGATC
	AAAGCGAATTTTAAAATCCGTCACAACGTGGAGGATGGGAGCGTGCAGTTAGCGGATCATTATCAGCAG
	AACACCCCGATTGGGGATGGGCCGGTGCTGCTGCCGGATAATCATTATCTGAGCACCCAATCCGTTCTG
	AGCAAAGATCCGAATGAAAAACGTGATCATATGGTGCTGCTGGAATTTGTTACCGCCGCGGGGATTACC
	CACGGGATGGATGAACTGTATAAA
GFP GGA	ATGAGCAAAGGTGAAGAACTGTTTACCGGCGTTGTGCCGATTCTGGTGGAACTGGATGGAGATGTGAAT
	GGACACAAATTTAGCGTTCGTGGAGAAGGAGAAGGAGATGCGACCAACGGAAAACTGACCCTGAAATTT
	ATTTGCACCACCGGAAAACTGCCGGTTCCGTGGCCCACCCTGGTGACCACCCTGACCTATGGAGTTCAG
	TGTTTTAGCCGCTATCCGGATCATATGAAACGCCATGATTTCTTTAAAAGCGCGATGCCGGAAGGATAT
	GTGCAGGAACGTACCATTAGCTTCAAAGATGATGGAACCTATAAAACCCGTGCGGAGGTGAAATTTGAA
	GGAGATACCCTCGTGAACCGCATTGAACTGAAAGGAATTGATTTTAAAGAAGATGGAAATATTCTGGGA
	CATAAACTGGAATATAATTTTAACAGCCATAATGTGTATATTACCGCCGATAAACAGAAAAATGGAATC
	AAAGCGAATTTTAAAATCCGTCACAACGTGGAGGATGGAAGCGTGCAGTTAGCGGATCATTATCAGCAG
	AACACCCCGATTGGAGATGGACCGGTGCTGCTGCCGGATAATCATTATCTGAGCACCCAATCCGTTCTG
	AGCAAAGATCCGAATGAAAAACGTGATCATATGGTGCTGCTGGAATTTGTTACCGCCGCGGGAATTACC
	CACGGAATGGATGAACTGTATAAA
GFP GAG	ATGAGCAAAGGTGAAGAACTGTTTACCGGCGTTGTGCCGATTCTGGTGGAGCTGGATGGTGATGTGAAT
	GGCCATAAATTTAGCGTTCGTGGCGAGGGAGAGGGAGATGCGACCAACGGTAAACTGACCCTGAAATTT
	ATTTGCACCACCGGTAAACTGCCGGTTCCGTGGCCCACCCTGGTGACCACCCTGACCTATGGCGTTCAGT
	GTTTTAGCCGCTATCCGGATCATATGAAACGCCATGATTTCTTTAAAAGCGCGATGCCGGAGGGCTATG
	TGCAGGAGCGTACCATTAGCTTCAAAGATGATGGCACCTATAAAACCCGTGCGGAGGTGAAATTTGAGG
	GCGATACCCTCGTGAACCGCATTGAGCTGAAAGGTATTGATTTTAAAGAGGATGGCAATATTCTGGGTC
	ATAAACTGGAGTATAATTTTAACAGCCATAATGTGTATATTACCGCCGATAAACAGAAAAATGGCATCA
	AAGCGAATTTTAAAATCCGTCACAACGTGGAGGATGGTAGCGTGCAGTTAGCGGATCATTATCAGCAGA
	ACACCCCGATTGGTGATGGCCCGGTGCTGCTGCCGGATAATCATTATCTGAGCACCCAATCCGTTCTGA
	GCAAAGATCCGAATGAGAAACGTGATCATATGGTGCTGCTGGAGTTTGTTACCGCCGCGGGCATTACCC
	ACGGTATGGATGAGCTGTATAAA
GFP CTA	ATGAGCAAAGGTGAAGAACTGTTTACCGGCGTTGTGCCGATTCTGGTGGAACTAGATGGTGATGTGAAT
	GGCCACAAATTTAGCGTTCGTGGCGAAGGAGAAGGAGATGCGACCAACGGTAAACTAACCCTAAAATTT
	ATTTGCACCACCGGTAAACTACCGGTTCCGTGGCCCACCCTAGTGACCACCCTAACCTATGGCGTTCAGT
	GTTTTAGCCGCTATCCGGATCATATGAAACGCCATGATTTCTTTAAAAGCGCGATGCCGGAAGGCTATG
	TGCAGGAACGTACCATTAGCTTCAAAGATGATGGCACCTATAAAACCCGTGCGGAGGTGAAATTTGAAG
	GCGATACCCTAGTGAACCGCATTGAACTAAAAGGTATTGATTTTAAAGAAGATGGCAATATTCTAGGTC
	ATAAACTAGAATATAATTTTAACAGCCATAATGTGTATATTACCGCCGATAAACAGAAAAATGGCATCA
	AAGCGAATTTTAAAATCCGTCACAACGTGGAGGATGGTAGCGTGCAGCTAGCGGATCATTATCAGCAGA
	ACACCCCGATTGGTGATGGCCCGGTGCTACTACCGGATAATCATTATCTAAGCACCCAATCCGTTCTAAG
	CAAAGATCCGAATGAAAAACGTGATCATATGGTGCTACTAGAATTTGTTACCGCCGCGGGCATTACCCA
	CGGTATGGATGAACTATATAAA
